# Structured reporting after sternotomy: a three-compartment approach

**DOI:** 10.1590/0100-3984.2025.0047

**Published:** 2025-08-01

**Authors:** André Vaz, Vinícius Cardoso Serra

**Affiliations:** 1 Instituto do Coração do Hospital das Clínicas da Faculdade de Medicina da Universidade de São Paulo (InCor/HC-FMUSP), São Paulo, SP, Brazil

We would like to congratulate Reifegerste et al.^**(1)**^ for their excellent review of the literature on imaging findings following sternotomy, published in **Radiologia Brasileira**. The article offers a comprehensive overview of the expected postoperative findings and complications following thoracic surgery. To address this clinically relevant and frequently encountered scenario, the use of a structured report would significantly enhance clarity and consistency, facilitating interdisciplinary communication and enabling improved clinical decision-making and follow-up.

We propose the use of a structured template that categorizes postoperative findings into three anatomical compartments^**(2-4)**^: presternal, sternal, and retrosternal. Each compartment may present with specific findings as follows (illustrative case in Figure 1).

**Figure 1:**
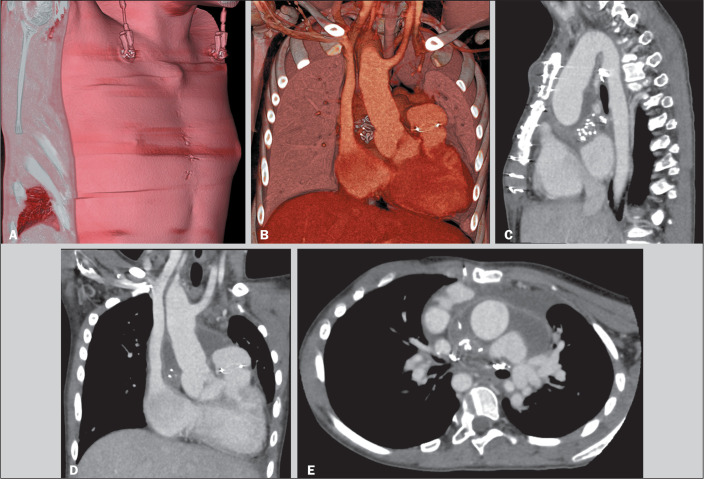
A 13-year-old girl with a history of pulmonary atresia and ventricular septal defect underwent corrective surgery. She developed postoperative fever and an increase in soft-tissue volume in the surgical wound. She was referred for contrast-enhanced chest computed tomography. Volumetric rendering of three-dimensional reconstructions (A,B), together with the sagittal, coronal, and axial images (C, D, and E, respectively), revealed the following in the presternal compartment: soft-tissue incision with opposing margins; and a subcutaneous fluid collection, measuring 8 mL, in the superior portion. The sternal compartment showed the median sternotomy with opposing margins and intact, aligned transverse transsternal sutures. In the retrosternal compartment, there was mild pericardial effusion with pericardial wall thickening and enhancement, together with a heterogeneous area containing radiopaque marker suggestive of retained surgical material, located on the right side, posterolateral to the ascending aorta. A second sternotomy revealed purulent mediastinitis with a retained gauze adjacent to the ascending aorta.

## Presternal compartment

*a) Closure:*
  - Soft tissue incision with opposing margins.
  - Soft tissue dehiscence.
*b) Content:*
  - Mild adipose tissue stranding related to recent manipulation/superficial sternal wound infection.
  - Superficial/deep subcutaneous fluid collection, measuring [ ] mL.
*c) Devices:*
  - Tubular drain with superficial/deep subcutaneous tip.
  - Vacuum-assisted soft tissue closure.

## Sternal compartment

*a) Closure:*
  - Median sternotomy with opposing margins.
  - Delayed sternal closure with fragments separated by [ ] mm.
  - Sternal dehiscence with bone fragments separated by [ ] mm at the upper, middle, or lower third.
*b) Content:*
  - Discrete irregularity of the sternal margins suggestive of reparative bone changes.
  - Osteolytic lesions at the sternal margins suggestive of osteomyelitis.
*c) Devices:*
  - Intact and aligned transverse peristernal/transsternal/figure-of-eight/Robicsek sutures/plates and screws.
  - Transverse peristernal/transsternal/figure-of-eight/Ro- --bicsek sutures/plates and screws with fracture/displacement in the upper/middle/lower third.

## Retrosternal compartment

*a) Content:*
  - Mild mediastinal adipose tissue stranding consistent with recent postoperative changes.
  - Persistent/progressive mediastinal adipose tissue stranding suggestive of deep sternal wound infection/mediastinitis.
  - Anterior/superior/anterosuperior mediastinal fluid collection defined as mild (< 10 mm), moderate (10-20 mm), or severe (> 20 mm).
  - Pericardial effusion defined as mild (< 10 mm), moderate (10-20 mm), or severe (> 20 mm).
  - Heterogeneous area containing radiopaque marker suggestive of retained surgical material, located in [ ].
*b) Devices:*
  - Mediastinal drainage tube in a retrosternal, paracardiac, retrocardiac, infracardiac, or supracardiac position.
  - Retrosternal synthetic membrane-assisted pericardial closure.
  - Temporary pacemaker with epicardial leads.

We believe that incorporating such a standardized format would not only enhance the clarity of radiologic reports but also facilitate better management of postoperative complications.

Once again, we commend Reifegerste et al.^**(1)**^ for their outstanding contribution and look forward to further advancements in the imaging of thoracic surgical patients.
